# Targeting the MLN64/STARD3-cholesterol axis disrupts tight junction remodelling and attenuates gastric cancer aggressiveness

**DOI:** 10.3389/fonc.2026.1913732

**Published:** 2026-10-15

**Authors:** Amber Xinyu Li, Yiran Chen, Xinguo Zhuang, Layla Alshammari, Elyas Khan, Q. Ping Dou, Tracey A. Martin, Wen G. Jiang

**Affiliations:** 1 Cardiff University School of Medicine, Cardiff, United Kingdom; 2 Karmanos Cancer Institute, Department of Oncology, School of Medicine, Wayne State University, Detroit, MI, United States; 3 Henry Wellcome Building for Biomedical Research, University Hospital of Wales, Cardiff, United Kingdom

**Keywords:** cholesterol trafficking, drug repurposing, gastric cancer, MLN64, therapeutic target, tight junction

## Abstract

**Introduction:**

Gastric cancer remains a major cause of cancer-related mortality, largely due to invasion, metastasis, and therapeutic resistance. Dysregulated cholesterol metabolism is increasingly recognised as a driver of gastric cancer progression, but how intracellular cholesterol trafficking contributes to malignant junctional remodelling remains poorly understood. MLN64/STARD3 is a cholesterol-binding lipid transfer protein associated with cancer progression, yet its functional role in gastric cancer cell architecture and aggressiveness has not been fully defined.

**Methods:**

In this study, we investigated whether MLN64 is associated with cholesterol-dependent tight junction organisation and malignant behaviour in gastric cancer cells.

**Results:**

Stable MLN64 knockdown in AGS and HGC-27 cells reduced membrane-associated free cholesterol, as shown by an approximately 50% decrease in the Filipin III membrane/cytoplasm fluorescence ratio in both cell lines. MLN64 depletion was also associated with reduced ZO-1 protein abundance, disrupted junctional ZO-1 localisation, and impaired tight junction integrity, including reduced transepithelial resistance, altered impedance, and increased paracellular permeability. Functionally, MLN64 depletion attenuated gastric cancer cell proliferation and invasive capacity, supporting an association between this cholesterol-trafficking axis, junctional organisation, and aggressive cellular phenotypes. To explore the pharmacological tractability of this pathway, in silico screening of an FDA-approved drug library identified Digoxin and Targretin as candidate compounds predicted to interact with the MLN64 START domain. Digoxin showed a favourable predicted binding energy of -13.73 kcal/mol. Functional validation showed that these agents phenocopied several effects of MLN64 depletion, including altered cholesterol distribution, reduced junctional ZO-1 organisation, impaired adhesion/migration dynamics, and reduced barrier function.

**Conclusion:**

Together, these findings identify MLN64 as a regulator associated with cholesterol-dependent tight junction remodelling in gastric cancer cells. Digoxin and Targretin provide preliminary evidence that this pathway may be pharmacologically tractable; however, direct target-engagement and rescue studies will be required to establish causality and determine whether their effects are mediated through direct interaction with MLN64. The MLN64- cholesterol axis may represent a potential vulnerability in gastric cancer membrane organisation and progression.

## 1 Introduction

Gastric cancer remains one of the leading causes of cancer-related mortality worldwide, with poor outcomes largely driven by late diagnosis, metastatic dissemination, and therapeutic resistance (1, 2). Despite advances in molecular classification and targeted therapy, effective treatment options for aggressive and recurrent gastric cancer remain limited. Increasing evidence suggests that metabolic reprogramming, particularly altered lipid and cholesterol metabolism, contributes to gastric cancer progression by supporting membrane organisation, oncogenic signalling, invasion, and treatment resistance (3). However, the molecular mechanisms linking intracellular cholesterol trafficking to the structural and functional remodelling of gastric cancer cells remain incompletely understood.

Cholesterol is not only a metabolic substrate but also a key architectural component of cellular membranes (4). By enriching lipid rafts and membrane microdomains, cholesterol regulates receptor signalling, cell adhesion, polarity, and junctional complex stability (5–9). In cancer cells, abnormal cholesterol accumulation and redistribution can promote malignant behaviour by sustaining growth-factor signalling, cytoskeletal remodelling, and invasive capacity (10). In gastric cancer, cholesterol-associated pathways have been linked to HER2 signalling, drug resistance, and metastatic progression, highlighting lipid transport and membrane cholesterol homeostasis as potential therapeutic vulnerabilities (1, 3, 11).

MLN64, also known as STARD3, is a member of the StAR-related lipid transfer protein family and contains a START domain capable of binding cholesterol. MLN64 is frequently co-amplified with ERBB2/HER2 and has been implicated in several malignancies, including breast, gastric, hepatic, and ovarian cancers (12–14). Functionally, MLN64 regulates intracellular cholesterol distribution through its localisation to late endosomes and its ability to mediate membrane contact sites, particularly between late endosomes and the endoplasmic reticulum (15, 16). Through these activities, MLN64 is thought to influence cholesterol mobilisation, lipid storage, and cancer-associated signalling pathways (17, 18). Nevertheless, its role in gastric cancer cell architecture and junctional regulation remains poorly defined.

Tight junctions are critical regulators of epithelial barrier function, polarity, and intercellular communication. The tight junction proteins can have context-dependent roles in cancer development and progression: loss of epithelial barrier integrity may facilitate dissemination, while selected junctional components can also support tumour cell survival, adhesion, signalling, and migration (19). ZO-1, encoded by TJP1, is a major scaffolding protein that links transmembrane tight-junction proteins to the actin cytoskeleton and helps organise junctional signalling platforms. Recent studies have shown that cholesterol-rich membrane domains are important for tight-junction assembly and ZO protein function, suggesting that altered cholesterol trafficking may directly influence junctional architecture in cancer cells (4, 5).

Although MLN64 is recognised as a cholesterol-trafficking protein and has been associated with cancer progression, whether it regulates cholesterol-dependent tight junction remodelling in gastric cancer remains unknown. We hypothesised that MLN64 supports gastric cancer aggressiveness by maintaining membrane cholesterol pools required for ZO-1 localisation, junctional organisation, and malignant cellular behaviour.

In this study, we investigated the role of MLN64/STARD3 in gastric cancer cell cholesterol distribution, tight junction integrity, and aggressive phenotypes using stable MLN64-knockdown AGS and HGC-27 cell models. We further explored the pharmacological tractability of this pathway through in silico screening of an FDA-approved drug library followed by functional validation of candidate compounds. Our findings identify MLN64 as a regulator of cholesterol-dependent tight junction remodelling and suggest that disruption of the MLN64-cholesterol axis may represent a potential strategy for targeting membrane-associated mechanisms of gastric cancer progression.

## 2 Materials and methods

### 2.1 Cell lines and reagents

Human gastric adenocarcinoma AGS and HGC-27 cells were obtained from the European Collection of Authenticated Cell Cultures (ECACC, Salisbury, UK). AGS cells were used as a primary gastric adenocarcinoma model, while HGC-27 cells were used as a metastatic gastric cancer model. Cells were maintained in Dulbecco’s Modified Eagle Medium (DMEM) supplemented with 10% foetal calf serum (FCS), 1% penicillin/streptomycin, and cultured at 37 °C in a humidified incubator containing 5% CO_2_.

Pharmacological agents including Digoxin (D6003) and Targretin (Bexarotene, SML0282) were purchased from Sigma-Aldrich (Gillingham, UK). For imaging and barrier assays, Filipin III solution (SAE0087) and FITC-Dextran 40 were utilised.

### 2.2 Generation of stable MLN64kd cell Lines

Stable MLN64/STARD3 knockdown cells were generated using lentiviral shRNA vectors targeting human MLN64. AGS and HGC-27 cells were transduced with pLV[shRNA]-EGFP:T2A:Puro-U6>hSTARD3 constructs, including two independent shRNA sequences, designated SH1 and SH2, or a non-targeting scrambled control vector. The constructs were obtained from VectorBuilder. The shRNA target sequences were: SH1: CAGGAAGAGAACTGGAAGTTT; SH2: GAGATCATCCAGTACAACTTT. Twenty-four hours after transduction, cells were selected with 2 μg/ml puromycin. Stable knockdown populations were maintained in medium containing 0.2 μg/ml puromycin. Knockdown efficiency was confirmed by qPCR and Western blotting before functional assays.

### 2.3 RNA extraction and qPCR analysis

RNA was extracted using TRI Reagent according to the manufacturer’s instructions (Sigma-Aldrich, UK). Gene expression was analysed by real-time quantitative PCR (qPCR) using the Amplifluor molecular beacon system. Primer sequences were as follows: MLN64 5’*gcacctttgtctggattctt’3 and 5’ actgaacctgaccgtacatgaaaggcaaattcaaacat’3, and GAPDH 5’aaggtcatccatgacaactt’3 and 5’actgaacctgaccgtacagccatccacagtcttctg’3.* (actgaacctgaccgtaca *for the* Z sequence to complement the FAM-tagged Uniprimer™ probe).

### 2.4 RNA sequencing, proteomic and phosphoproteomic analysis

For transcriptomic analysis, total RNA was extracted from scrambled control and MLN64-knockdown AGS and HGC-27 cells. RNA quality and integrity were assessed by Biomarker Technologies (Münster, Germany), and transcriptomic analysis was performed according to the manufacturer’s standard protocols.

For proteomic and phosphoproteomic analysis, protein lysates from scrambled control and MLN64-knockdown cells were prepared using RIPA lysis buffer. Proteomic and phosphoproteomic profiling was performed by the University of Bristol Proteomics Facility. Protein identification and quantification were carried out against the human UniProt database using the facility’s standard analysis pipeline.

### 2.5 Western blotting

Cells were lysed in RIPA lysis buffer supplemented with protease and phosphatase inhibitors. Protein concentration was determined using the BCA assay. Equal amounts of protein were separated by SDS-PAGE on gels and transferred to PVDF membranes.

Membranes were blocked with 10% non-fat milk in TBS with 0.1% tween (T) for 1 hour at room temperature and incubated overnight at 4 °C with primary antibodies against MLN64/STARD3 (SC-390040, 1:500), ZO-1 (clone 1A12, Invitrogen, 1:500), and loading control GAPDH (SC-32233, 1:1500). Membranes were then incubated with HRP-conjugated secondary antibody for 1 hour at room temperature (anti-mouse IgG: A5278, 1:2000; anti-rabbit IgG: A9169).

Bands were visualised using enhanced chemiluminescence with Westar Antares substrate and imaged using a chemiluminescence G:BOX imaging (Syngene, Cambridge, UK) system. Densitometric analysis was performed using ImageJ. Target protein levels were normalised to the corresponding loading control and expressed relative to scrambled control cells.

### 2.6 Paracellular permeability (PCP)

Paracellular permeability was assessed using FITC-dextran 40 kDa. Cells were seeded onto 0.4 μm pore-size transwell inserts at a density of 5 x10^4^ cells per insert and grown until confluent monolayers were established. FITC-dextran was added to the upper chamber at 2 mg/mL with or without drug treatment. Medium from the lower chamber was collected over 24 hours.

Fluorescence intensity was measured using a Glomax multiwell fluorescence reader. Paracellular flux was calculated relative to untreated scrambled control monolayers.

### 2.7 Transepithelial electrical resistance (TER/TEER)

Transepithelial electrical resistance was measured using an EVOM voltohmmeter equipped with STX-2 chopstick electrodes. Cells were seeded onto 0.4 μm at 5 x10^4^ density and cultured until confluent. Resistance was measured at baseline and at indicated time points following MLN64 knockdown or drug treatment. The resistance values were multiplied by the membrane surface area to obtain Ω·cm². Data were expressed as absolute resistance values or normalised to baseline/control conditions.

### 2.8 Electric cell-substrate impedance sensing (ECIS)

Electric cell-substrate impedance sensing was used to monitor cell adhesion, migration, and barrier-associated impedance in real time. ECIS 96W1E arrays were stabilised, activated, and coated according to the manufacturer’s instructions. Cells were seeded at a 5x10^4^ cells density per well in complete medium, with or without drug treatment.

Impedance was recorded using an ECIS system from Applied Biophysics. For adhesion assays, impedance was monitored immediately after seeding and normalised to the time-zero or untreated control value. For migration assays, confluent monolayers were electrically wounded and recovery of impedance over time was used as a measure of migration. For barrier assays, impedance of confluent monolayers was recorded over time.

### 2.9 Transwell invasion assay

Cell invasion was assessed using transwell chambers with Matrigel-coated inserts with 0.8 µm pores. For Matrigel assays, inserts were coated with 5.0 µg/well and allowed to polymerise before cell seeding. Cells were seeded into the upper chamber at 50000 in serum-free medium. Complete medium containing 20% FCS was added to the lower chamber as a chemoattractant.

After 72 hours, non-invading cells were removed from the upper membrane surface. Invaded cells on the lower surface were fixed with 4% formalin, stained with 10% crystal violet, and counted under microscope. Invasion was normalised to proliferation data at the corresponding time point to account for differences in cell growth.

### 2.10 Immunofluorescence and Filipin III cholesterol staining

Cells were seeded onto 10% FCS-coated chamber slides and incubated overnight prior to treatment with the indicated compounds for 4 hours. Cells were fixed with 4% paraformaldehyde, permeabilised using 0.01% Saponin, and blocked with 5% horse serum in PBS. ZO-1 was detected using primary antibodies (1:500) and fluorescence-conjugated secondary antibodies (1:1500), together with Filipin III (20 μg/ml) for co-visualisation of ZO-1 and free cholesterol. Slides were mounted with FluoSave™ and imaged using an Olympus fluorescence microscope equipped with a Hamamatsu ORCA-ER camera.

Filipin III fluorescence was quantified using ImageJ/Fiji to assess the subcellular distribution of free cholesterol. Images were acquired using identical exposure settings within each experiment and analysed without post-acquisition intensity adjustment. For each cell, membrane-associated regions of interest were defined along the plasma membrane or cell-cell junctional border, while cytoplasmic regions of interest were selected within the same cell, avoiding the nucleus, saturated puncta, and cell-free areas. Background fluorescence was measured from cell-free regions of each image and subtracted from all membrane and cytoplasmic measurements.

Mean fluorescence intensity was recorded for each region of interest (n=9). The membrane/cytoplasm fluorescence ratio was calculated for each analysed cell using the formula: Membrane/cytoplasm ratio = background-corrected membrane mean intensity/background-corrected cytoplasmic mean intensity.

### 2.11 Cholesterol quantification

Cellular cholesterol was quantified using the Cholesterol/Cholesteryl Ester Assay Kit (ab65359, Abcam) according to the manufacturer’s instructions. Briefly, 1×10^6^ cells were extracted in chloroform:isopropanol:NP-40 (7:11:0.1), and the organic phase was collected following centrifugation at 13,000×g for 10 minutes. Lipid extracts were dried, resuspended in Cholesterol Assay Buffer, and sonicated until homogenous. Total cholesterol was measured following cholesterol esterase treatment, while free cholesterol was assessed in parallel reactions without esterase. Cholesterol levels were determined colorimetrically at OD570 nm, and cholesteryl ester content was calculated by subtracting free cholesterol from total cholesterol. Values were normalised to total protein content determined by BCA assay.

### 2.12 Virtual screening of FDA drug library for MLN64/STARD3-interacting candidate

The crystal structure of the human MLN64 lutein/cholesterol-binding domain (amino acids F231–R444; PDB: 5I9J) was obtained from the RCSB database and prepared using BIOVIA Discovery Studio Visualizer (BIOVIA, 2021) by removing water molecules, heteroatoms, and bound ligands. Virtual screening of an FDA-approved drug library containing 1615 compounds from the ZINC database was performed using PyRx (20) against the prepared MLN64 structure with a grid box encompassing the full protein. The top 50 compounds with the lowest binding energies were subsequently validated using AutoDock (21). Based on binding affinity and reproducibility, Digoxin and Targretin were selected for further biochemical and cell-based validation assays.

### 2.13 Statistical analysis

Statistical analyses were performed using GraphPad Prism version 9.0. Data are presented as mean ± SD from at least three independent biological experiments unless otherwise stated. Comparisons between two groups were performed using two-tailed Student’s t-test. Multiple-group comparisons and time-course experiments were analysed using one-way ANOVA or repeated-measures ANOVA where measurements were obtained from the same experimental units over time. A p-value < 0.05 was considered statistically significant. Significance was indicated as *p < 0.05, **p < 0.01, and ***p < 0.001.

## 3 Results

### 3.1 MLN64 depletion suppresses gastric cancer cell proliferation and invasion

To determine the functional role of MLN64 in gastric cancer progression, we first assessed the effects of stable MLN64 knockdown in AGS and HGC-27 cells (**Figure 1A**). MLN64 silencing significantly reduced cell proliferation in both gastric cancer cell lines over a five-day period, with a clear divergence from scrambled control cells becoming evident from day 3 and increasing by day 5. These findings indicate that MLN64 contributes to sustained proliferative capacity in gastric cancer cells.

**Figure 1:**
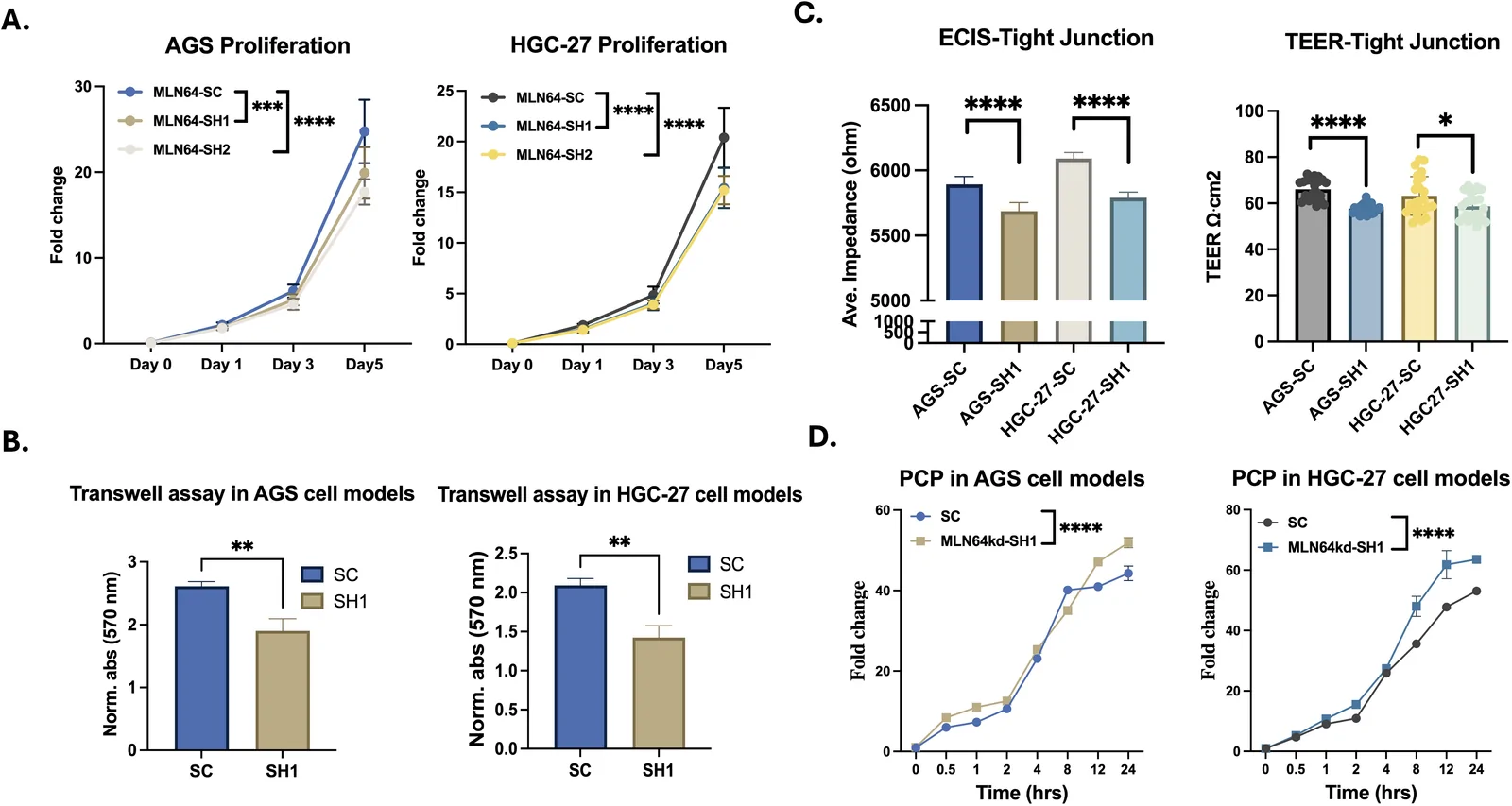
MLN64 silencing inhibits proliferation and invasion and impairs tight junction integrity and in gastric cancer cells. **(A)** Growth assays revealed that stable MLN64 knockdown (SH1 and SH2) significantly reduced proliferation in AGS and HGC-27 cells over 5 days. **(B)** Transwell invasion assays showed that MLN64 silencing markedly suppressed the invasive capacity of both gastric cancer cell lines. **(C)** ECIS and TEER analyses showed significantly reduced impedance and resistance in MLN64-silenced (SH1) AGS and HGC-27 cells compared to scrambled controls (SC), indicating impaired tight junction integrity. **(D)** PCP assays demonstrated increased FITC-dextran flux in MLN64-knockdown cells, confirming enhanced epithelial permeability. (*Data are presented as mean ± SD from at least three independent experiments. Statistical significance was determined using Student’s t-test or repeated-measures ANOVA (*p < 0.05, **p < 0.01, and ***p < 0.001.).

We next examined whether MLN64 regulates invasive behaviour (**Figure 1B**). Transwell invasion assays showed that MLN64 depletion markedly reduced the invasive capacity of both AGS and HGC-27 cells. To account for differences in proliferation, invasion was normalised to the corresponding growth data at day 3. The reduction in invasion after MLN64 knockdown suggests that MLN64 supports aggressive gastric cancer cell behaviour beyond its effects on cell growth.

### 3.2 MLN64 depletion compromises epithelial barrier function and tight junction integrity

Because tight junction remodelling can influence gastric cancer cell behaviour, we next examined whether MLN64 regulates epithelial barrier properties (**Figure 1C**). ECIS analysis of confluent monolayers showed that MLN64-silenced cells had reduced impedance compared with scrambled controls, indicating impaired junctional integrity. Consistently, transepithelial electrical resistance was significantly decreased in MLN64-knockdown cells, confirming reduced barrier resistance.

Paracellular permeability assays using FITC-dextran further demonstrated that MLN64 depletion increased tracer flux across gastric cancer cell monolayers (**Figure 1D**). Together, these findings show that MLN64 is required for the maintenance of tight junction-associated barrier function in AGS and HGC-27 cells.

### 3.3 MLN64 depletion is associated with reduced ZO-1 protein abundance and disrupted junctional localisation

To define the molecular basis of MLN64-dependent barrier regulation, we examined tight junction-associated proteins. RNA sequencing revealed altered expression of the *Zona Occludens (ZO/TJP)* family following MLN64 knockdown, with significant reductions in *TJP2* and *TJP3*, while *TJP1 (ZO-1)* transcripts remained largely unchanged in both AGS and HGC-27 cells (**Figure 2A**). qPCR further confirmed that *ZO-1* mRNA expression was not significantly altered (**Figure 2B**).

**Figure 2:**
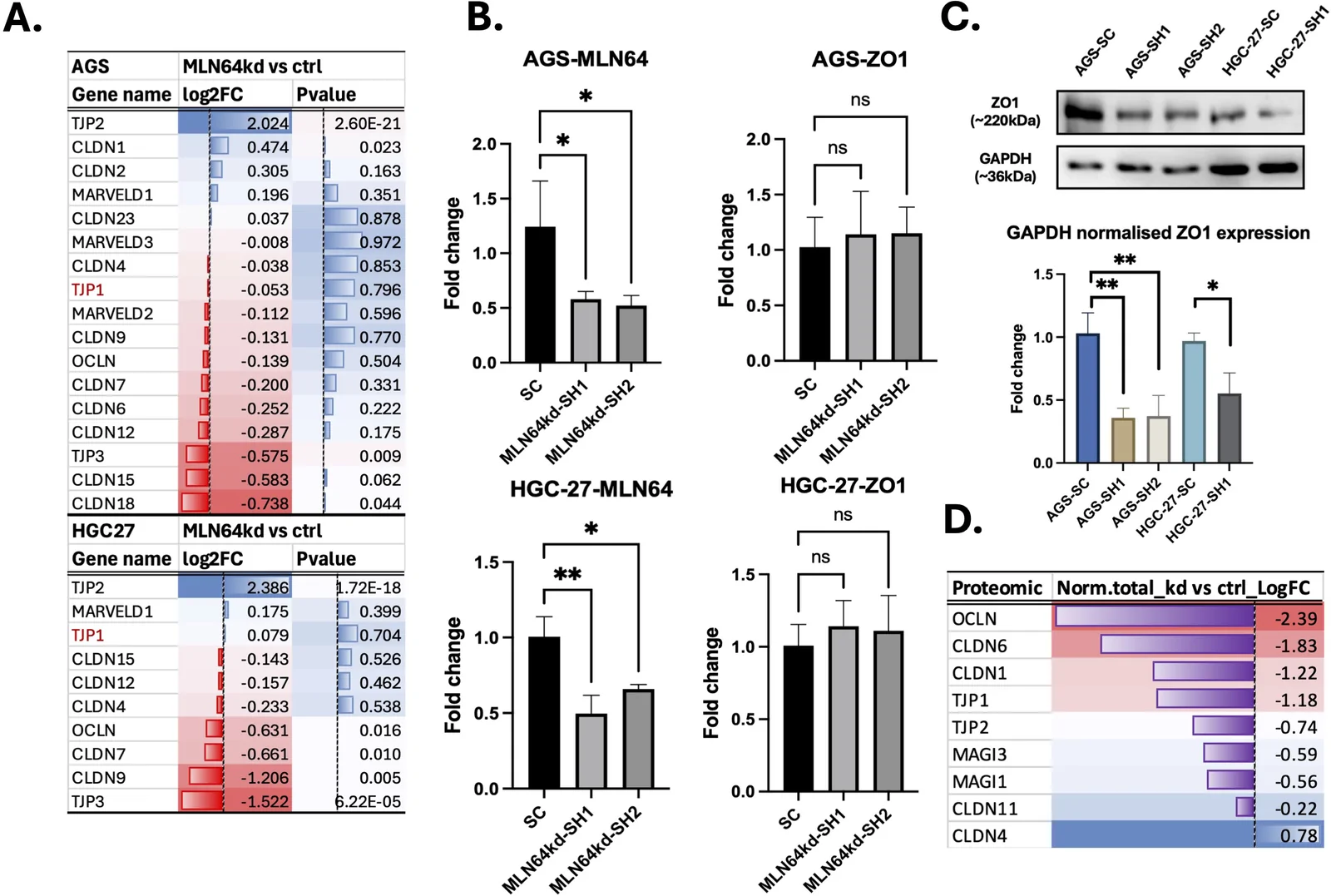
MLN64 regulates expressions of tight junctions proteins. **(A)** Heatmaps showing transcriptomic changes in tight junction-associated genes following MLN64 knockdown in AGS and HGC-27 cells. Alterations were observed in claudin and TJP family members. **(B)** RT-qPCR validation confirming MLN64 knockdown (SH1, SH2), while ZO-1 mRNA levels remained unchanged. **(C)** Western blot analysis demonstrating reduced ZO-1 protein expression in MLN64-deficient cells. **(D)** Proteomic profiling revealed decreased expression of key tight junction proteins, including OCLN, CLDN6, and TJP1 (ZO-1). *p < 0.05, **p < 0.01.

In contrast, Western blotting showed a marked reduction in ZO-1 protein abundance in MLN64-deficient cells. (**Figure 2C**). Proteomic analysis further supported this finding, revealing an approximately 10-fold decrease in total ZO-1 protein levels (Log2FC = −1.18) in MLN64-deficient cells (**Figure 2D**).

Immunofluorescence microscopy further revealed disruption of ZO-1 junctional localisation. Whereas control cells displayed continuous ZO-1 staining at cell-cell borders, MLN64-knockdown cells (**Figure 3A**) showed fragmented and punctate ZO-1 staining with reduced membrane localisation (**Figure 3B**).

**Figure 3:**
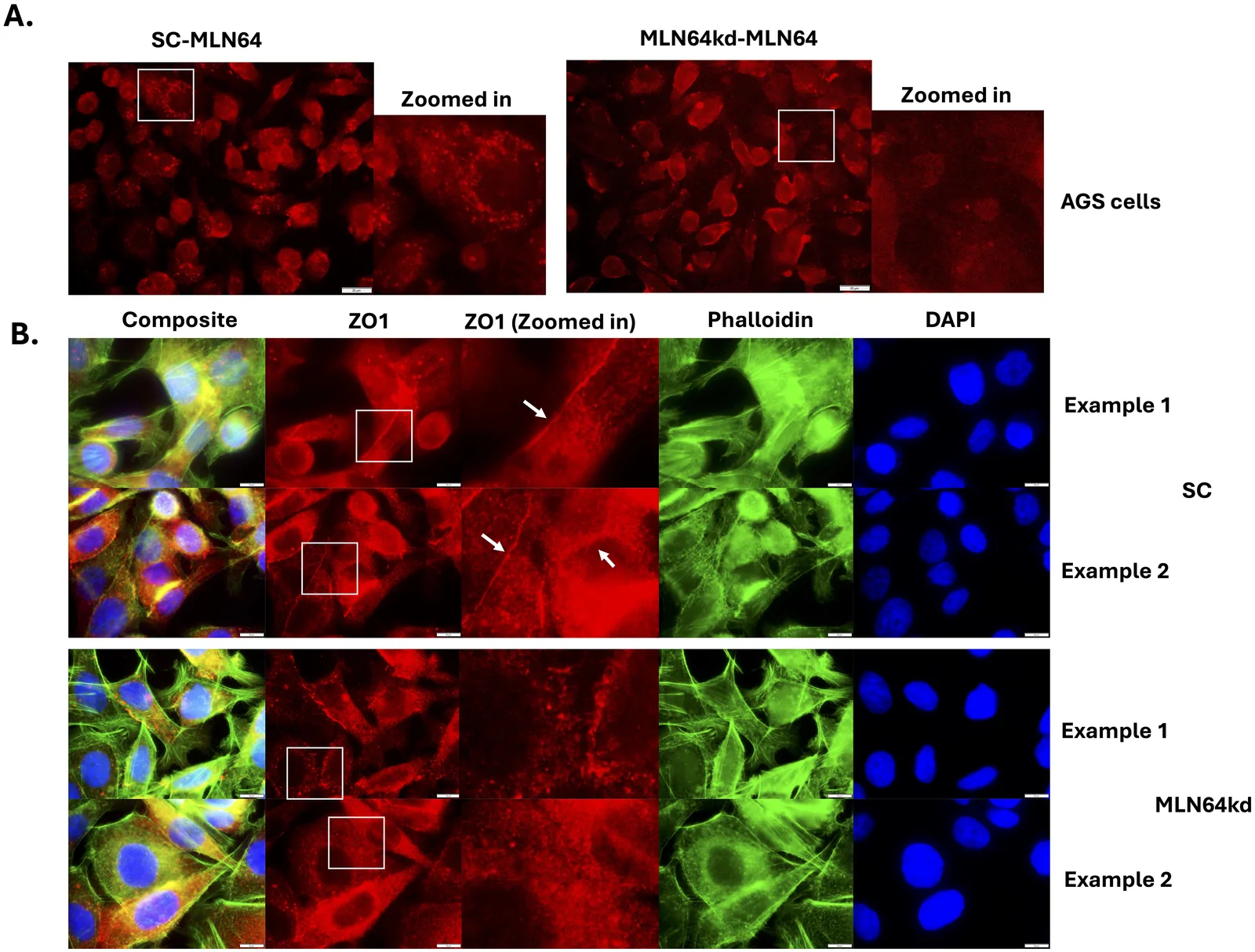
Immunofluorescence imaging of MLN64 and the tight-junction protein ZO-1 in MLN64-knockdown cell models. **(A)** Representative immunofluorescence images confirming reduced MLN64 (red dots) expression in MLN64kd AGS cells compared with its corresponded SC cells. **(B)** Representative immunofluorescence images showing disrupted membrane localisation of ZO-1 in MLN64kd AGS cells compared with the continuous junctional staining observed in SC cells. ZO-1 is shown in red, phalloidin-labelled actin in green, and DAPI-stained nuclei in blue. Scale bars: 20 µm for the 40× images and 10 µm for the 100× magnified images.

To determine whether MLN64 depletion caused a broad collapse of tight junction components or preferentially affected the ZO scaffolding complex, we analysed tight junction-associated proteins using proteomic and phosphoproteomic profiling. Although total Occludin abundance was reduced (log_2_FC = -2.39), phosphorylation at S15 was increased (log_2_FC = 2.18), suggesting that tight junction remodelling after MLN64 depletion was not uniform across all components. In contrast, ZO-1 and ZO-2 showed reductions in both total protein abundance and phosphorylation at selected regulatory sites, including ZO-1 S3 and ZO-2 S3, S5, and Y3.

These findings suggest that MLN64 depletion is associated with altered ZO-family protein regulation at the post-transcriptional level. Because phosphorylation can influence ZO-1 junctional recruitment, scaffolding interactions, and protein turnover, reduced ZO-family phosphorylation may contribute to the observed loss of ZO-1 membrane localisation. However, additional mechanistic studies will be required to determine the exact mechaim.

### 3.4 MLN64 depletion alters membrane cholesterol distribution

Given the established role of MLN64 in intracellular cholesterol trafficking, we next investigated whether loss of MLN64 affects cholesterol distribution in gastric cancer cells. Filipin III staining showed strong membrane-associated free cholesterol enrichment in control AGS and HGC-27 cells. (**Figure 4**). In contrast, MLN64-knockdown cells exhibited reduced cholesterol signal at the plasma membrane, accompanied by a more diffuse or punctate intracellular cholesterol distribution. Quantitative analysis confirmed that the membrane/cytoplasm Filipin III fluorescence ratio was significantly reduced in both SH1 and SH2 MLN64-knockdown cells compared with scrambled controls in AGS and HGC-27 models (**Figure 4**). These results indicate that MLN64 is required for maintaining membrane-associated cholesterol pools in gastric cancer cells. The concurrent loss of membrane cholesterol and disruption of ZO-1 localisation suggests that MLN64-mediated cholesterol trafficking supports the lipid environment required for tight junction scaffolding (**Figure 3B**).

**Figure 4:**
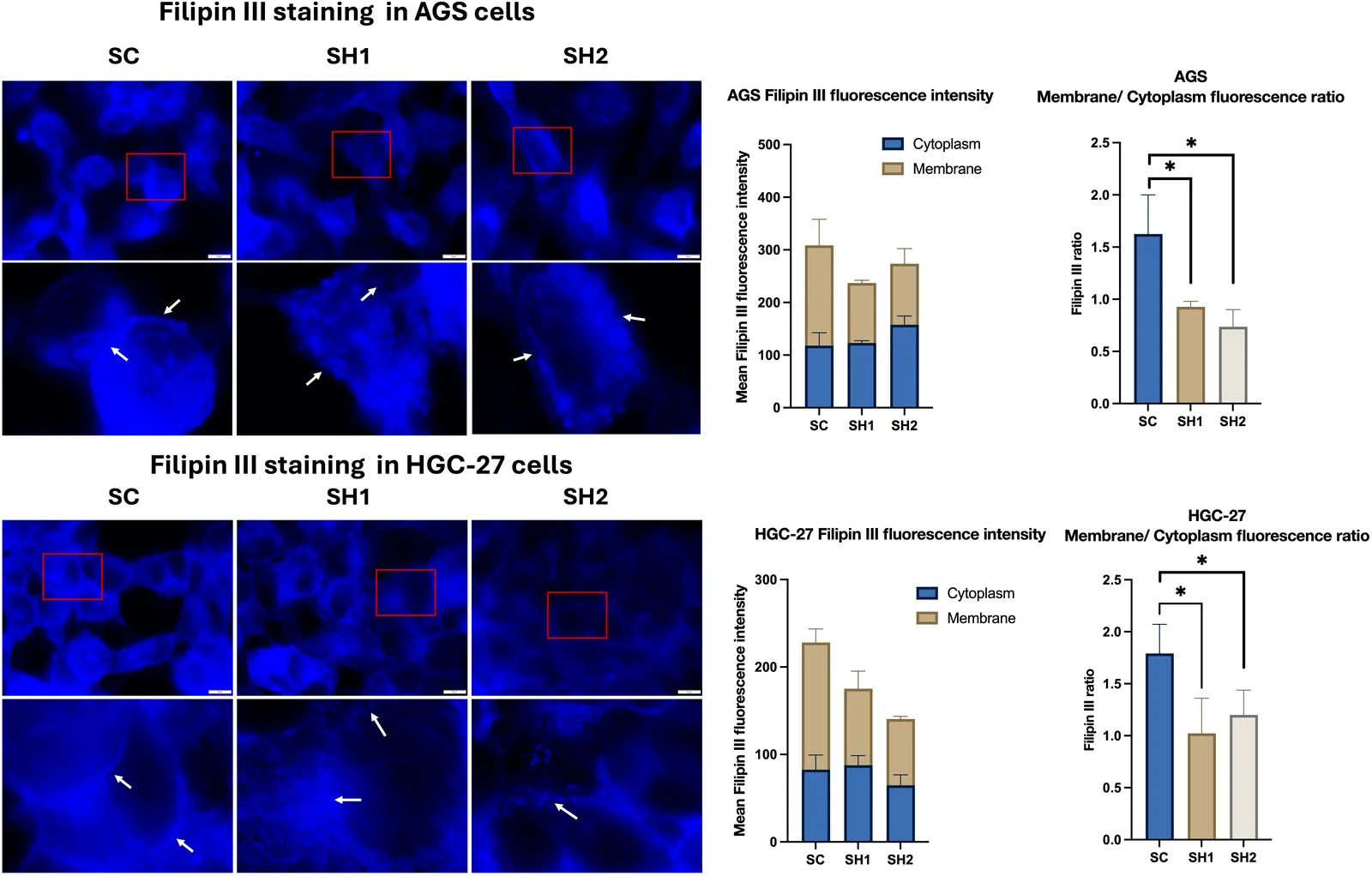
MLN64 depletion disrupts membrane-associated free cholesterol distribution in gastric cancer cells. Filipin III staining was used to visualise free cholesterol distribution in scrambled control (SC) and MLN64-silenced (SH1 and SH2) AGS and HGC-27 cells. Quantification of background-corrected Filipin III fluorescence intensity was performed in cytoplasmic and membrane-associated regions of interest, and the membrane/cytoplasm fluorescence ratio was calculated. Data are presented as mean ± SD. Statistical significance was determined using Student’s t-test or one-way ANOVA as appropriate. *p < 0.05, **p < 0.01. 10 µm for 100× magnified images. The zoomed panels are cropped from original fields.

### 3.5 Virtual screening identifies Digoxin and Targretin as candidate MLN64-targeting compounds

To identify candidate compounds predicted to interact with the MLN64/STARD3 START domain, we performed virtual screening of an FDA-approved drug library containing 1,615 compounds. The crystal structure of the human MLN64/STARD3 cholesterol-binding domain (PDB: 5I9J) was selected as the docking model and analysed using PyRx. This generated a report containing binding energies of all the drugs. We then selected the top 50 drugs with the most negative binding energy for further validation in AutoDock, and this practice resulted in a short list of top hits that have the most negative binding energies and greater reproducible histograms. Of these top hits, Digoxin and Targretin were selected and subjected for biological validation (**Figure 5**).

**Figure 5:**
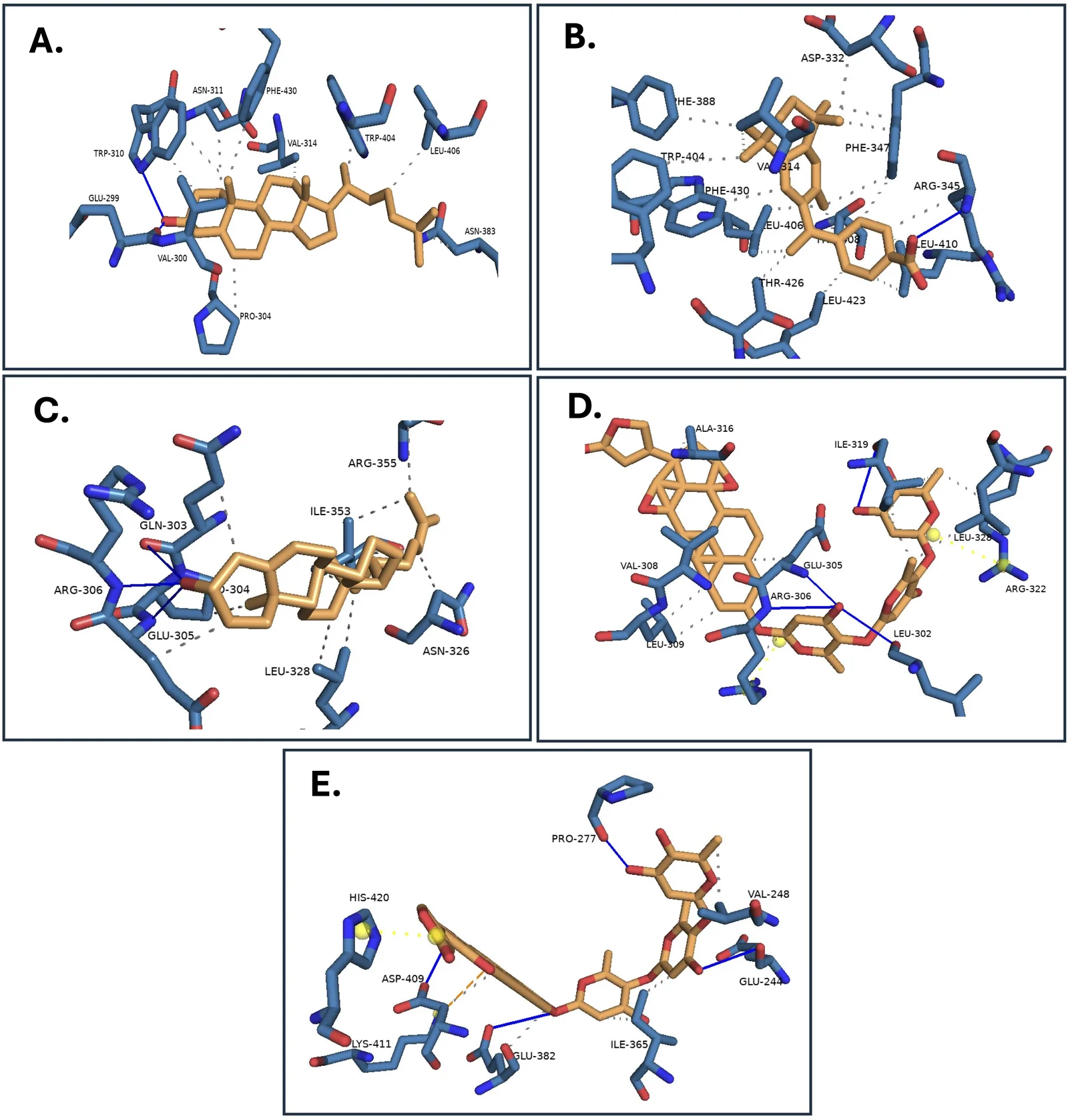
3D docking results between Targretin and Digoxin to MLN64 and cholesterol. **(A)** Conformation 1 of cholesterol docked to MLN64 (PDB: 5I9J). **(B)** Top-ranked conformation of Targretin docked to MLN64 (PDB: 5I9J). Targretin and cholesterol both interact with residues 314, 404, 406, and 430 of MLN64 via hydrophobic interactions. Grey dashes represent hydrophobic interactions, and blue lines represent hydrogen bonds. **(C)** Conformation 2 of cholesterol docked to MLN64 (PDB: 5I9J). **(D)** Conformation 1 of digoxin docked to MLN64 (PDB: 5I9J). Both digoxin and cholesterol interact with residues 305 and 306 via hydrogen bonding and 328 via hydrophobic interaction. Grey dashes represent hydrophobic interactions, and blue lines represent hydrogen bonds. **(E)** Most stable conformation of digoxin docked to MLN64 forms 4 hydrogen bonds (with 244, 277, 382, and 409), 1 pi-cation interaction with 411, 1 salt bridge 420, and 5 hydrophobic interactions (with 248, 365, 382, and 409). Grey dashes represent hydrophobic interactions, blue lines represent hydrogen bonds, orange dashes represent pi-cation interactions, and yellow spheres represent salt bridge contact points.

### 3.6 Digoxin and Targretin phenocopy MLN64 knockdown in cholesterol distribution, ZO-1 localisation, and functional assays

Lapillo et al., showed that cholesterol binds to MLN64 at a binding pocket formed by amino acids 313, 314, 335, 337, 347, 349, 351, 362, 388, 404, 406, 408, 410, 426, and 430 (18). Our docking of cholesterol to MLN64 gave a similar result, involving residues 299, 300, 304, 310, 311, 314, 383, 404, 406, 430 (**Figure 5A**, Cholesterol, Conformation 1), with amino acids 314, 404, 406 and 430 identical to the cholesterol-binding residues reported by Lapillo et al. (2019) (18).

Interestingly, our docked conformations showed that Targretin interacts with amino acids 314, 404, 406, and 430 via hydrophobic interactions (**Figure 5B**, Targretin), and these 4 residues are identical to the cholesterol-binding residues of MLN64 reported by Lapillo et al. (18). In addition, Targretin forms a hydrogen bond with amino acid 345 of MLN64 and a hydrophobic interaction with amino acid 332, both of which have been reported to be important for cholesterol binding (22).

Another docking conformation shows that cholesterol binds to a different binding pocket of MLN64 involving amino acids 303-306, 326, 328, 353 and 355 (**Figure 5C**, Cholesterol, Conformation 2). Our docking of Digoxin gave a conformation showing interaction with amino acids 302, 305, 306, 308, 309, 316, 319, 322, and 328, sharing amino acids 305, 306 (hydrogen bonding), and 328 (hydrophobic interaction) cholesterol binds to (**Figure 5D**, Digoxin, Conformation 1) in a cholesterol-mimicking mode.

The most stable conformation of Digoxin we identified shows that the drug binds tightly to MLN64 with a binding energy of -13.73 kcal/mol (**Figure 5E**, Digoxin, Conformation 2), suggesting potential potent inhibition. In this binding mode, Digoxin forms a number of stable contacts with MLN64, including 5 hydrophobic interactions, 4 hydrogen bonds, 1 pi-cation interaction, and 1 salt bridge, all of which contribute to the stable and tight binding of digoxin to MLN64.

### 3.7 Digoxin and Targretin phenocopy MLN64 depletion in gastric cancer cell adhesion and migration assays

We next tested whether Digoxin and Targretin reproduce functional effects associated with MLN64 loss. ECIS-based adhesion assays showed that both compounds reduced adhesion-associated impedance in control gastric cancer cells (**Figure 6A**). Digoxin produced a dose-dependent reduction in adhesion, while Targretin also impaired adhesion dynamics. These effects were reduced or absent in MLN64-knockdown cells, suggesting that the activity of these compounds may depend, at least in part, on the presence of functional MLN64.

**Figure 6:**
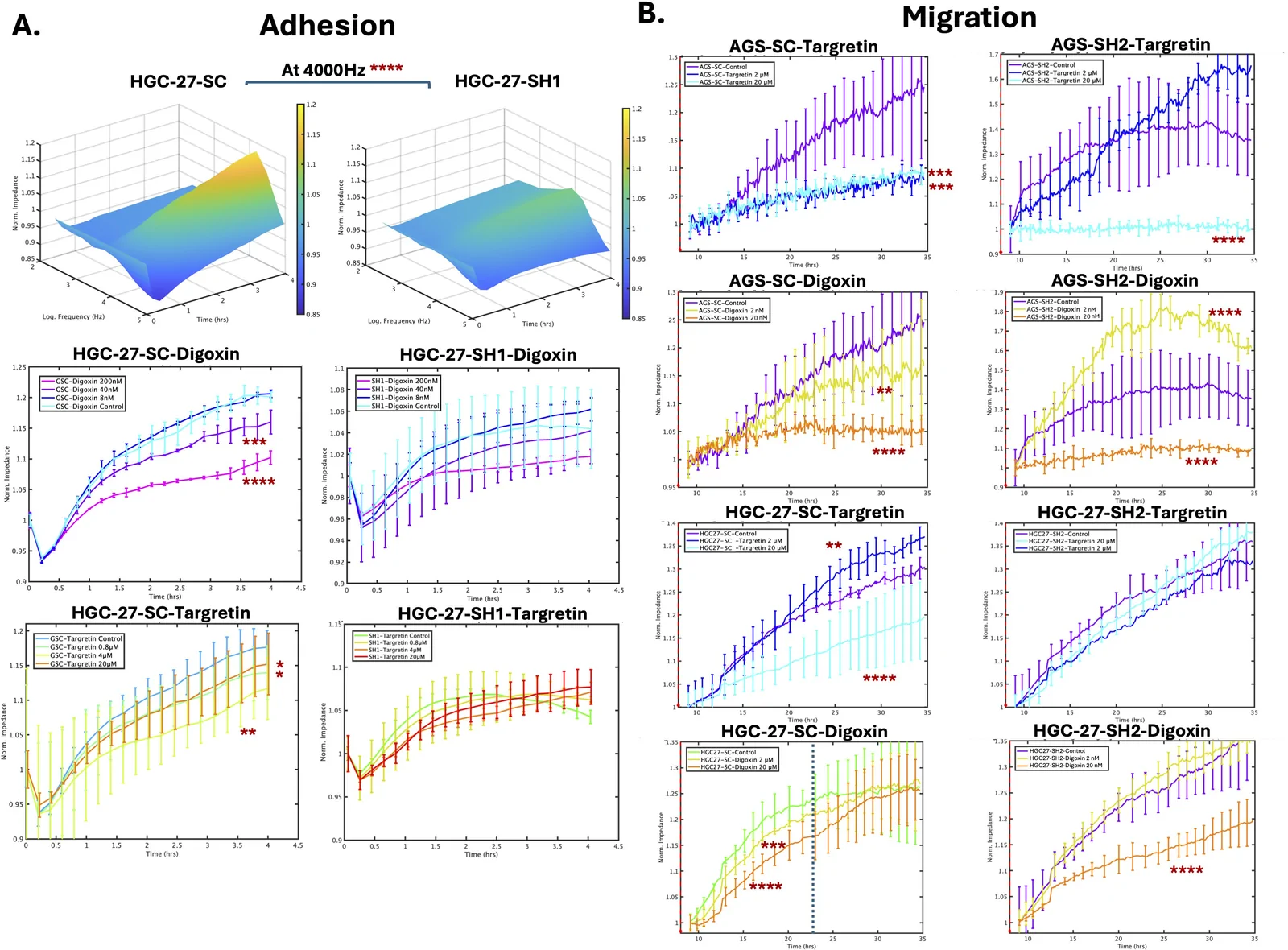
Digoxin and Targretin alter gastric cancer cell adhesion and migration. **(A)** 3D ECIS plots demonstrate reduced adhesion-related impedance in HGC-27 MLN64kd (SH1) cells compared to controls. Digoxin and Targretin treatment induced a dose-dependent reduction in adhesion in control cells. **(B)** ECIS migration assays showed that both Digoxin and Targretin significantly suppressed migration in AGS and HGC-27 gastric cancer cells. Statistical significance compared to untreated controls: **p < 0.01, ***p < 0.001 and **** p < 0.0001.

Migration assays further showed that Digoxin and Targretin suppressed gastric cancer cell migration, although sensitivity differed between AGS and HGC-27 cells (**Figure 6B**). Digoxin produced a dose-dependent inhibitory effect in both cell lines, while Targretin showed stronger activity in AGS cells at lower concentrations and required higher concentrations in HGC-27 cells. These findings support the functional relevance of the MLN64-cholesterol axis in regulating gastric cancer cell adhesion and motility.

### 3.8 Pharmacological perturbation of MLN64 alters cholesterol homeostasis and ZO-1 organisation

Quantitative cholesterol analysis showed that MLN64 knockdown and pharmacological treatment reduced free cholesterol and total cholesterol levels, with associated changes in cholesteryl ester pools (**Figure 7A**). These findings suggest that disruption of MLN64-mediated cholesterol trafficking affects both membrane cholesterol availability and broader intracellular cholesterol homeostasis.

**Figure 7:**
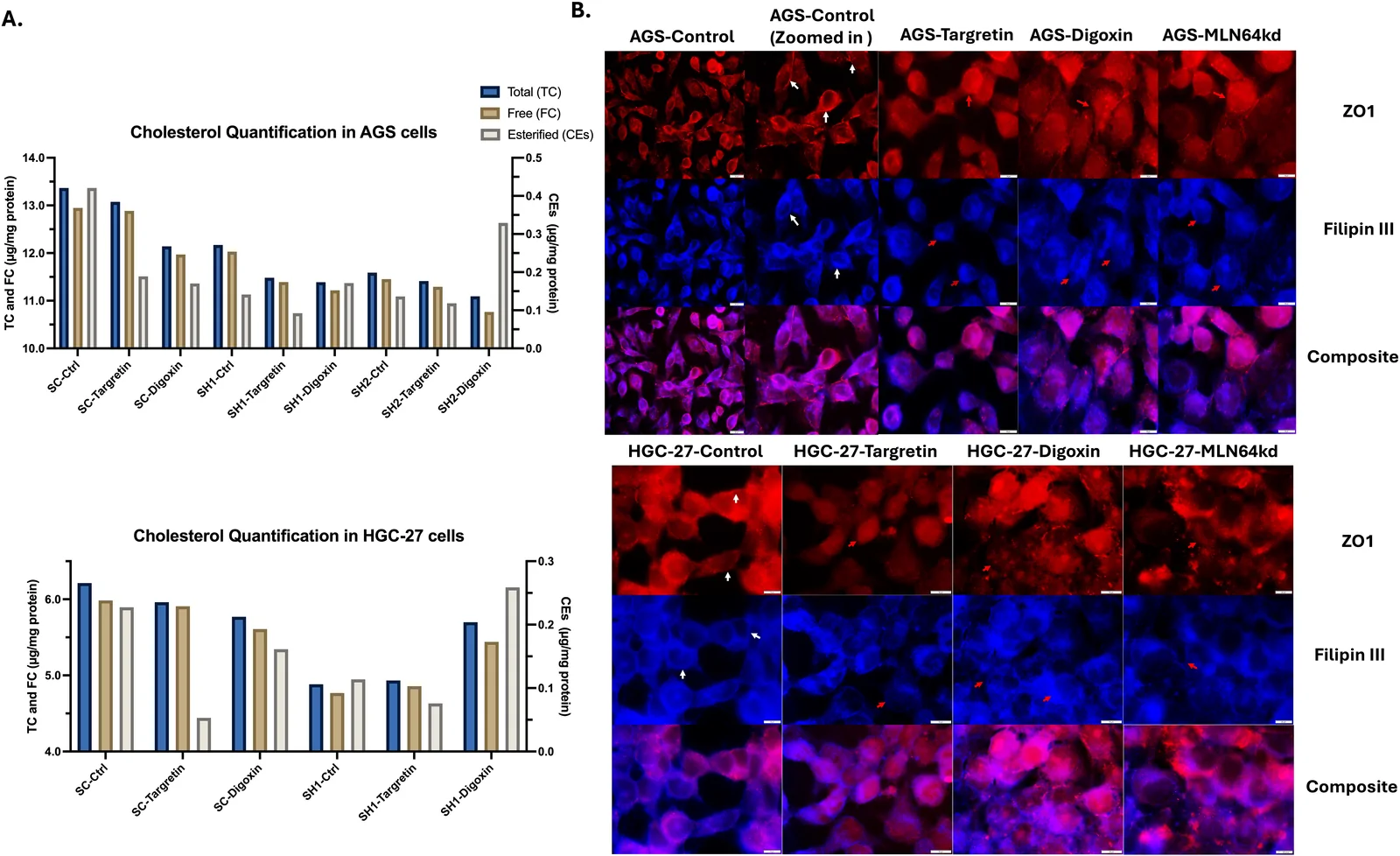
Digoxin and Targretin disrupt MLN64-mediated cholesterol homeostasis and ZO-1 localisation. **(A)** Quantitative analysis of total cholesterol (TC), free cholesterol (FC), and cholesteryl esters (CEs) in AGS and HGC-27 cells. MLN64 knockdown and treatment with Digoxin or Targretin reduced TC, FC, and CE levels compared to controls. **(B)** Immunofluorescence co-localisation of ZO-1 (red) and free cholesterol (Filipin III, blue). Control cells displayed strong junctional co-localisation, whereas MLN64 inhibition or knockdown caused loss of membrane cholesterol and fragmented ZO-1 localisation, indicating disrupted tight junction assembly. *Data are presented as mean ± SD. *p < 0.05, **p < 0.01, and ***p < 0.001. Scale bars: 20 µm for 40× images and 10 µm for 100× magnified images. The zoomed panels are cropped from original fields.

Immunofluorescence analysis demonstrated that drug-induced cholesterol redistribution was accompanied by loss of junctional ZO-1 localisation (**Figure 7B**). Control cells displayed clear co-localisation of free cholesterol and ZO-1 at cell-cell junctions, whereas Digoxin or Targretin treatment caused reduced membrane cholesterol signal and fragmented ZO-1 staining. These results indicate that pharmacological disruption of the MLN64-cholesterol axis impairs the cholesterol-dependent membrane environment required for ZO-1 junctional organisation.

### 3.9 Digoxin and Targretin impair tight junction barrier function

Finally, we assessed whether pharmacological disruption of the MLN64-cholesterol axis affects epithelial barrier function (**Figure 8**). ECIS analysis showed that Targretin reduced monolayer impedance in control cells in a dose-dependent manner, while the additional effect was attenuated in MLN64-knockdown cells (**Figure 8A**). Similarly, paracellular permeability assays demonstrated increased FITC-dextran flux following Digoxin or Targretin treatment, consistent with impaired tight junction integrity (**Figure 8B**).

**Figure 8:**
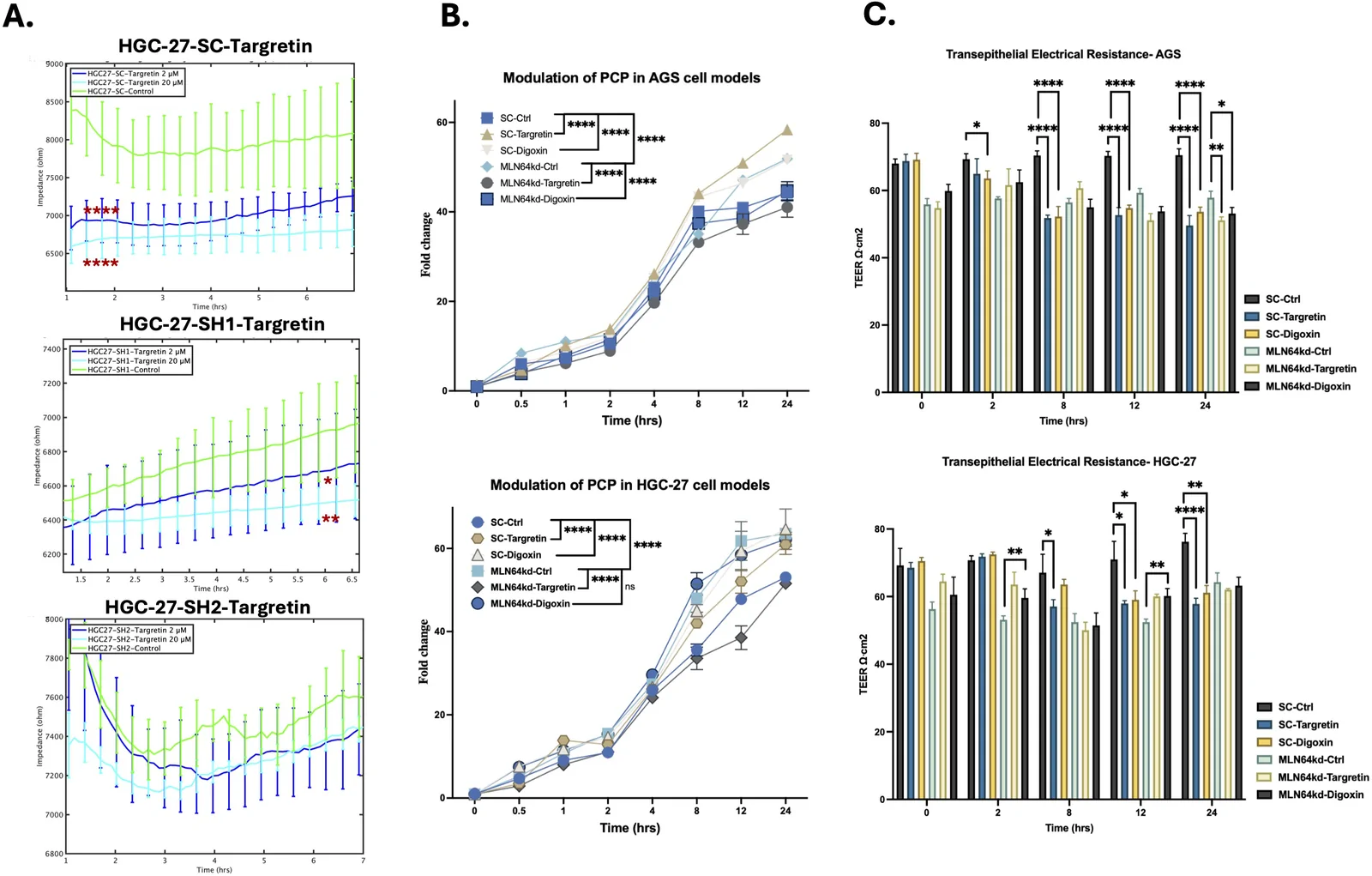
Pharmacological inhibition of MLN64 impairs gastric epithelial barrier function. **(A)** ECIS impedance analysis of HGC-27 SC, SH1, and SH2 cells treated with Targretin (2 μM and 20 μM). Targretin induced a rapid dose-dependent reduction in impedance in SC cells, while this effect was attenuated in MLN64kd models. **(B)** PCP assays demonstrated increased paracellular permeability following Digoxin or Targretin treatment in AGS and HGC-27 SC cells. Enhanced permeability was also observed in AGS MLN64kd cells, but was less pronounced in HGC-27 knockdown models. **(C)** TEER measurements over 24 hours showed that MLN64 knockdown significantly reduced barrier resistance compared to scrambled controls. Digoxin and Targretin further decreased TEER in SC cells, particularly at 8 and 24 hours, confirming impaired epithelial barrier integrity following disruption of MLN64-mediated cholesterol trafficking. Data are presented as mean ± SD. *p < 0.05, **p < 0.01, and **** p < 0.0001.

TEER measurements further confirmed that MLN64 depletion and pharmacological treatment reduced barrier resistance over time (**Figure 8C**). Together, these findings show that genetic and pharmacological disruption of MLN64 converges on a common phenotype characterised by altered cholesterol distribution, loss of junctional ZO-1 organisation, impaired barrier function, and reduced gastric cancer cell aggressiveness.

### 3.10 Clinical association of STARD3 and TJP1 expression in gastric cancer datasets

To assess the clinical relevance of the MLN64-ZO-1 axis in gastric cancer, publicly available gastric cancer datasets were analysed (**Figure 9**). High STARD3 expression was significantly associated with poorer overall survival, first progression survival, and post-progression survival. In contrast, high ZO-1 expression was associated with improved overall survival and post-progression survival, with a trend toward improved first progression survival. Analysis of TCGA-STAD (stomach adenocarcinoma) samples further showed that STARD3 expression was significantly higher in primary gastric tumours than in normal gastric tissues, whereas ZO-1 expression did not differ significantly between tumour and normal samples (**Figure 9**).

**Figure 9:**
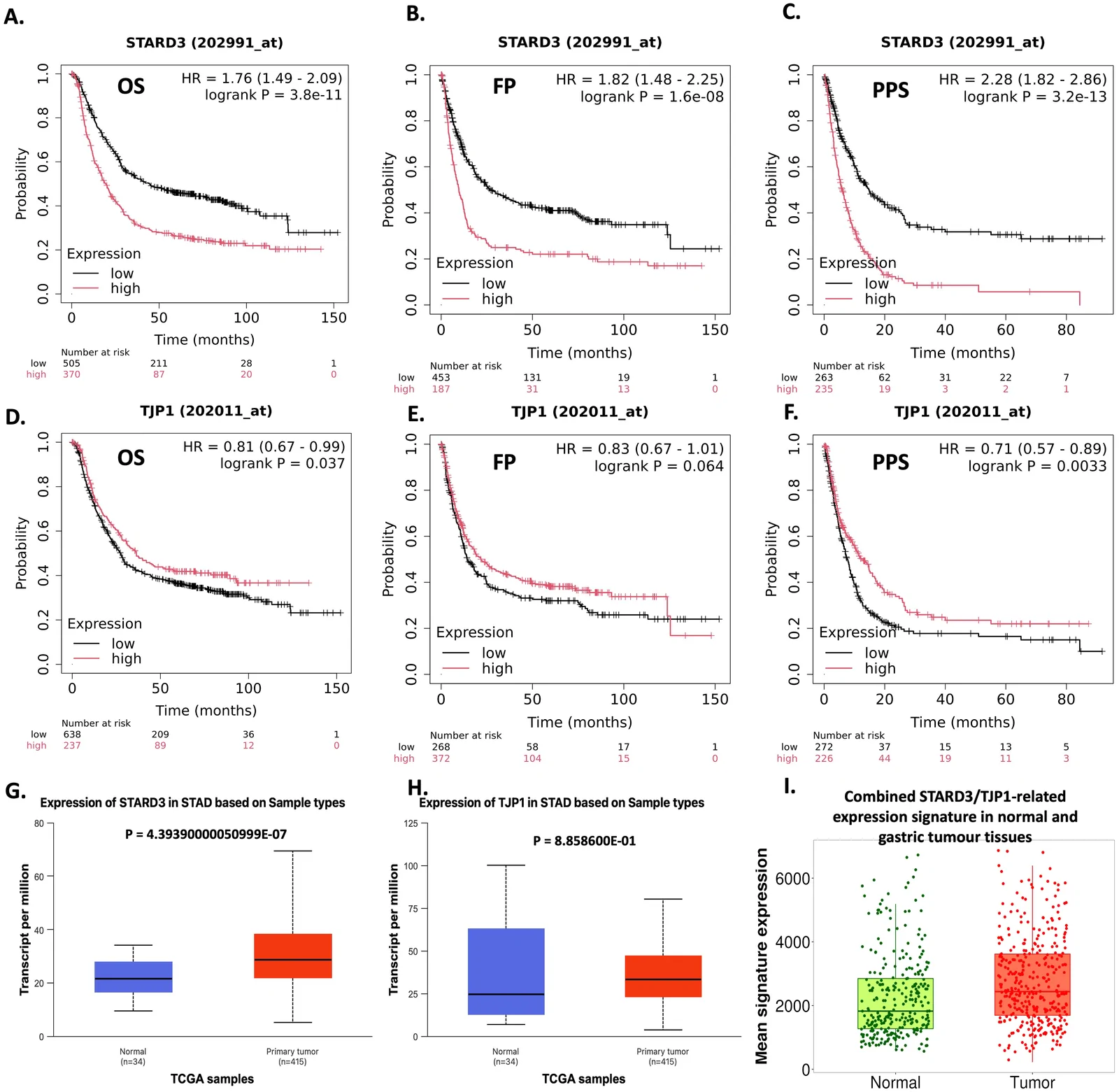
Clinical association of MLN64 and ZO-1 transcript expression in gastric cancer. Kaplan-Meier survival analysis was performed to assess the association between MLN64 (STARD3) and ZO-1 (TJP1) transcript expression and gastric cancer patient outcomes. High MLN64 expression was significantly associated with poorer overall survival [OS; **(A)**], first progression survival [FP; **(B)**], and post-progression survival [PPS; **(C)**]. In contrast, high ZO-1 expression was associated with improved overall survival **(D)** and post-progression survival **(F)**, with a trend toward improved first progression survival **(E)**. TCGA-STAD expression analysis showed that MLN64 expression was significantly higher in primary gastric tumour tissues than in normal gastric tissues **(G)**, whereas ZO-1 expression did not differ significantly between tumour and normal tissues **(H)**. Combined MLN64/ZO-1-related signature expression was also elevated in gastric tumour tissues compared with normal tissues **(I)**. Hazard ratios (HRs), 95% confidence intervals, and log-rank p-values are shown in each Kaplan-Meier plot. Expression data are presented as transcript per million. OS, overall survival; FP, first progression survival; PPS, post-progression survival; STAD, stomach adenocarcinoma.

Correlation analysis did not identify a significant association between STARD3 and TJP1 transcript levels in gastric cancer datasets. This suggests that the relationship between MLN64 and ZO-1 is unlikely to be mediated primarily through direct transcriptional regulation of TJP1, consistent with our *in vitro* findings showing altered ZO-1 protein abundance and localisation despite limited change in ZO-1 mRNA. Together, these analyses support the clinical relevance of STARD3 in gastric cancer and suggest that STARD3-associated cholesterol trafficking and ZO-1-associated junctional integrity may have distinct but clinically meaningful associations with patient outcome.

## 4 Discussion

Gastric cancer remains a major cause of cancer-related mortality worldwide, with disease progression driven by invasion, metastatic dissemination, and therapeutic resistance (2). Increasing evidence indicates that altered cholesterol metabolism contributes to gastric cancer progression by regulating membrane organisation, lipid raft-associated signalling, and drug response (3, 9). In this study, we identify MLN64 as a regulator of cholesterol-dependent tight junction remodelling in gastric cancer cells. Genetic silencing of MLN64 disrupted membrane-associated cholesterol distribution, reduced ZO-1 protein expression and junctional localisation, impaired epithelial barrier function, and attenuated proliferation and invasion in AGS and HGC-27 cells.

MLN64 has previously been implicated in cancer progression and is frequently associated with ERBB2/HER2-amplified malignancies (10, 11). In gastric cancer, genes co-amplified with ERBB2 or MET have been proposed to contribute to tumour-promoting phenotypes (11), while our previous work linked MLN64 expression to gastric cancer progression and drug resistance (1). The present study extends these observations by suggesting that MLN64 does not merely act as a cancer-associated marker but contributes functionally to the membrane cholesterol architecture required for gastric cancer cell behaviour.

Mechanistically, MLN64 is well positioned to regulate this process because it contains a START domain capable of binding cholesterol (12, 22) and participates in intracellular lipid trafficking through late endosomal compartments and membrane contact sites (16, 23). Our findings show that MLN64 depletion reduced membrane-associated free cholesterol and promoted intracellular redistribution of cholesterol, supporting the view that MLN64 helps maintain cholesterol availability at functionally important membrane regions. This is consistent with previous evidence that membrane lipid composition influences tight junction formation (4), and that cholesterol-rich domains mediated by ZO proteins are essential for tight junction assembly (6).

A major finding of this study is that MLN64/STARD3 loss reduced ZO-1 protein abundance and disrupted its junctional localisation despite limited changes in TJP1 transcript expression. These findings suggest that MLN64/STARD3 depletion affects ZO-1 at a post-transcriptional level, although the precise mechanism, including altered protein stability, degradation, phosphorylation-dependent recruitment, or membrane localisation, remains to be determined. Because ZO-1 functions as a scaffolding protein that links tight junction complexes to the actin cytoskeleton, disruption of cholesterol-rich membrane domains may destabilise ZO-1-containing junctional platforms and compromise barrier integrity.

Importantly, MLN64 depletion increased paracellular permeability and reduced barrier resistance, but also suppressed proliferation and invasion. These findings suggest that MLN64 does not simply preserve normal epithelial barrier function in gastric cancer cells. Rather, MLN64 appears to sustain a cholesterol-rich membrane architecture required for malignant cell adhesion, junctional scaffolding, motility, and invasion. Therefore, disruption of MLN64 weakens barrier integrity while simultaneously collapsing membrane-associated signalling and structural platforms that gastric cancer cells require for aggressive behaviour.

This interpretation is supported by the observed changes in additional tight junction components. Reduced expression of claudin family members may be particularly relevant, as Claudin-1 and Claudin-6 have been implicated in gastric cancer progression through pathways involving β-catenin and YAP/SNAIL1 signalling (25, 26). Although reduced ZO-1 expression has often been associated with tumour dissemination in other cancer types (24), our data indicate that, in these gastric cancer models, MLN64-dependent junctional architecture may support rather than suppress malignant behaviour.

We also explored whether the MLN64-cholesterol axis could be pharmacologically perturbed. Previous work identified VS1 as a first-in-class STARD3 inhibitor with anti-cancer activity (18). In the present study, virtual screening identified Digoxin and Targretin as candidate compounds predicted to interact with the MLN64 cholesterol-binding domain. Functional assays showed that these agents phenocopied several effects of MLN64 depletion, including altered cholesterol distribution, impaired ZO-1 localisation, reduced adhesion/migration dynamics, and weakened barrier function. However, these findings should be interpreted cautiously, as docking and phenotypic similarity do not prove direct MLN64 target engagement.

Targretin, an FDA-approved retinoid X receptor agonist, has been reported to suppress cancer cell viability and migration through pathways including SLC10A2/PPARγ/PTEN/mTOR signalling (25). Interestingly, Targretin has also been linked to improved endothelial barrier integrity through ApoE-associated effects on tight junction proteins (26, 27). The apparent contrast with our findings may reflect cellular context. In gastric cancer cells, where cholesterol trafficking may be rewired to support malignant membrane organisation, perturbation of the MLN64-cholesterol axis may destabilise rather than restore junctional architecture.

Digoxin also has recognised anti-cancer activity through mechanisms involving Na+/K+-ATPase, HIF-1α, and NF-κB signalling (28). Recent evidence further suggests that Digoxin can promote anoikis in metastatic gastric cancer cells (29), consistent with earlier observations that cholesterol depletion can induce anoikis-like apoptosis through FAK downregulation and caveolae internalisation (30). Since MLN64 has previously been linked to adhesion and FAK-associated phenotypes (14), future studies should determine whether MLN64 inhibition promotes anoikis or disrupts adhesion survival signalling in gastric cancer.

Although our data support a link between MLN64 depletion, cholesterol redistribution, ZO-1 disruption, and altered gastric cancer cell behaviour, the precise molecular mechanism connecting MLN64-dependent lipid trafficking to ZO-1 stability and junctional localisation remains to be fully defined. A further limitation is the absence of rescue experiments. Although our knockdown and pharmacological data support an association between MLN64 depletion, altered cholesterol distribution, impaired ZO-1 localisation, and barrier dysfunction, MLN64 re-expression, ZO-1 rescue, and cholesterol supplementation studies will be required to establish direct causality. These studies are currently being pursued as part of ongoing mechanistic work. Finally, Digoxin and Targretin have established targets outside MLN64/STARD3, including pathways related to mTOR, HIF-1α, NF-κB, and Na+/K+-ATPase (25, 28). Direct target-engagement studies, cholesterol-binding competition assays, rescue experiments, and MLN64 overexpression models will therefore be required to confirm whether these compounds act directly through MLN64.

Finally, this study was performed primarily in two-dimensional gastric cancer cell models. Although these systems are useful for defining cell-intrinsic mechanisms, they do not fully capture the complexity of the tumour microenvironment, stromal interactions, extracellular matrix organisation, or *in vivo* lipid availability. Future work should validate the MLN64-cholesterol-ZO-1 axis in three-dimensional cultures, organoid models, animal systems, and clinically annotated gastric cancer specimens.

In conclusion, our findings identify MLN64 as a regulator of cholesterol-dependent tight junction remodelling in gastric cancer cells. By maintaining membrane-associated cholesterol pools, MLN64 supports ZO-1 junctional localisation, barrier-associated architecture, and aggressive cellular phenotypes. Digoxin and Targretin functionally perturb several cholesterol-dependent phenotypes associated with MLN64 depletion, suggesting that this pathway may be pharmacologically tractable. However, direct target-engagement and rescue studies will be required to determine whether these compound effects are mediated through direct interaction with MLN64/STARD3. Together, these results suggest that the MLN64-cholesterol axis represents a potential vulnerability in gastric cancer membrane organisation and progression.

## Data Availability

The original contributions presented in the study are publicly available. This data can be found here: https://www.ebi.ac.uk/pride/archive; PXD085415, https://www.ncbi.nlm.nih.gov/geo/; GSE350741.

## Glossary

AGS: Human Gastric Adenocarcinoma Cell Line
ANOVA: Analysis of Variance
ApoE: Apolipoprotein E
BCA: Bicinchoninic Acid
CE: Cholesteryl Ester
CO₂: Carbon Dioxide
DMEM: Dulbecco’s Modified Eagle Medium
ECIS: Electric Cell–Substrate Impedance Sensing
EMT: Epithelial–Mesenchymal Transition
ER: Endoplasmic Reticulum
ERBB2/HER2: Erb-B2 Receptor Tyrosine Kinase 2
FAK: Focal Adhesion Kinase
FC: Free Cholesterol
FCS: Fetal Calf Serum
FITC: Fluorescein Isothiocyanate
GAPDH: Glyceraldehyde-3-Phosphate Dehydrogenase
GC: Gastric Cancer
HGC-27: Human Gastric Carcinoma Cell Line
HIF-1α: Hypoxia-Inducible Factor 1 Alpha
HSP: Heat Shock Protein
IF: Immunofluorescence
kDa: Kilodalton
LDL: Low-Density Lipoprotein
MCS: Membrane Contact Sites
MENT: MLN64 N-terminal Domain
MLN64: Metastatic Lymph Node 64
mTOR: Mammalian Target of Rapamycin
NF-κB: Nuclear Factor Kappa B
NHERF1: Na+/H+ Exchange Regulatory Factor 1
NSCLC: Non-Small Cell Lung Cancer
PCP: Paracellular Permeability
PI3K: Phosphoinositide 3-Kinase
PPARγ: Peroxisome Proliferator-Activated Receptor Gamma
qPCR: Quantitative Polymerase Chain Reaction
RXR: Retinoid X Receptor
SC: Scrambled Control
SD: Standard Deviation
SF: Serum-Free
shRNA: Short Hairpin RNA
STARD3: StAR-Related Lipid Transfer Domain Containing 3
START: StAR-Related Lipid Transfer
TEER: Transepithelial Electrical Resistance
TJ: Tight Junction
TJP: Tight Junction Protein
TC: Total Cholesterol
TME: Tumour Microenvironment
VAP: Vesicle-Associated Membrane Protein-Associated Protein
TJ-1/ZO-1: Tight Junction protein-1/Zonula Occludens-1
TJ-2/ZO-2: Tight Junction protein-2/Zonula Occludens-2
TJ-3/ZO-3: Tight Junction protein-3/Zonula Occludens-3.
